# POIS, a Low Cost Tilt and Position Sensor: Design and First Tests

**DOI:** 10.3390/s150510806

**Published:** 2015-05-07

**Authors:** Giuseppe Artese, Michele Perrelli, Serena Artese, Sebastiano Meduri, Natale Brogno

**Affiliations:** 1 Civil Engineering Department, University of Calabria, Via Bucci Cubo 45B, 87036 Rende, Italy; E-Mails: michele.perrelli@unical.it (M.P.); natalebrogno@tiscali.it (N.B.); 2 Department of Informatics, Modeling, Electronic and System Engineering—DIMES, University of Calabria, Via P. Bucci Cubo 42C, 87036 Rende, Italy; E-Mails: serena.artese@unical.it (S.A.); sebastiano.meduri@gmail.com (S.M.)

**Keywords:** tilt sensor, optoelectronic level, GNSS, wireless, landslide monitoring, structure monitoring

## Abstract

An integrated sensor for the measurement and monitoring of position and inclination, characterized by low cost, small size and low weight, has been designed, realized and calibrated at the Geomatics Lab of the University of Calabria. The design of the prototype, devoted to the monitoring of landslides and structures, was aiming at realizing a fully automated monitoring instrument, able to send the data acquired periodically or upon request by a control center through a bidirectional transmission protocol. The sensor can be released with different accuracy and range of measurement, by choosing bubble vials with different characteristics. The instrument is provided with a computer, which can be programmed so as to independently perform the processing of the data collected by a single sensor or a by a sensor network, and to transmit, consequently, alert signals if the thresholds determined by the monitoring center are exceeded. The bidirectional transmission also allows the users to vary the set of the monitoring parameters (time of acquisition, duration of satellite acquisitions, thresholds for the observed data). In the paper, hardware and software of the sensor are described, along with the calibration, the results of laboratory tests and of the first in field acquisitions.

## 1. Introduction

Tilt and positioning sensors have been always used for monitoring landslides and structures [1,2]. The use of geodetic methods for this purpose can be tracked back to its roots in Switzerland in the early years of the 20th century [3,4]. The inclination measurement and the positioning are generally obtained in an independent manner. For landslides, borehole inclinometers are used to detect the depth of the sliding surfaces and the direction of movement. On the surface, tilt measurements are performed mainly for the control of equipment. 

The techniques used for tilt measurement are numerous, and in recent years several optoelectronic instruments, based on various operating principles, have been realized. Tools based on the use of a commercial Digital Versatile Disc burner (DVD) pickup head as the angle sensor have been created [1]. Other instruments make use of light refraction in a transparent and viscous fluid, along with a reflection; such sensors use a light source and photodiodes for the reception of the returning beams [2,3,4,5,6]. More sophisticated sensors have been conceived and implemented in high-end instruments, like the Leica TS30 total station; in this case, the dual axis inclination sensor mainly consists of an oil layer in a casing (liquid prism) together with a prism and mirror, a prism with line patterns, a one-dimensional line sensor, and a light source [7]; actually, the liquid prism had been used since several years by inventors for tilt measurements [8,9].

A pendulum, along with mirrors and a 2D Position Sensing Detector, are on the basis of other realizations [10]. Also the shadow of a small suspended mass, projected onto a Complementary Metal-Oxide Semiconductor based (CMOS-based) optical sensor surface, can be exploited [11]. For better performances, the use of fiber optic is widespread [12,13], while the electrolytic sensors seem to be a promising technique to obtain both accuracy and wide range of measurements [14]. Very common is the use of inclinometers based on Micro Electro-Mechanical Systems (MEMS) accelerometers; these instruments are characterized, in general, by reduced dimensions and weight. Some models have very good performances, and they are used for realizing sensors for structural monitoring [15]. Very often MEMS accelerometers are joined with gyroscopes and low cost Global Navigation Satellite System (GNSS) receivers or Global Positioning System (GPS) receivers, to obtain navigation systems [16].

With regard to the positioning systems for landslide monitoring, the use of GNSS receivers has been a common practice for several years [17]. Low end receivers are suitable for utilization, but there are no examples showing both very low cost and accuracy: actually, in several recent applications, it is foreseen the use of Original Equipment Manufacturer (OEM) receivers, whose cost is not really low [18]; in other cases, a permanent stations network [19] or a reference station [20] is needed. Anyway, a center for data processing and transmission is foreseen, for applying the Real Time Kinematic (RTK) method.

Recently, low cost tilt sensors, based on MEMS technology, have been put on the market for monitoring landslides; these instruments are put in place on poles driven into the ground [21]. These sensors have limited precision; therefore, they are used primarily for the detection of sudden movements, precursors of the critical phases of the landslide, and for the activation of alarms. 

Much wider is the panorama of sensors for the monitoring of structures, with the possibility of choice of range and precision of measurements.

An ideal sensor for landslide monitoring, should have the following characteristics: (a) low cost, in order to have the possibility to use several sensors for each slope; (b) small size and weight; (c) tilt measurement in two directions; (d) selectable range and accuracy, depending on the landslide velocity; (e) possibility of working stand-alone or as network node; (f) possibility to install the sensor in the ground or of fixing it on the rock or on a structure; (g) position measurement; (h) data processing capability and (i) bidirectional data connection, in order to allow data transmission to a monitoring center and to receive commands for changing monitoring parameters. 

The increasing diffusion of credit-card sized, single board computers, like Arduino^®^ or Raspberry^®^, offers the possibility to manage different sensors and to integrate them in a single instrument, capable of autonomous data processing and of bidirectional transmission. 

In the following, an integrated wireless sensor for the measurement and monitoring of position and inclination, is described. The instrument, named Position and Inclination Sensor (POIS), is characterized by low cost, small size, low weight, low power consumption and it has been designed, realized and calibrated at the Geomatics Lab of the University of Calabria. 

With reference to the above described characteristics of an ideal sensor, the POIS sensor displays the following features: (a) the cost of the hardware components is about 400 $, thus the final price could be one order of magnitude less than that of the current high-end electronic instruments; (b) the dimensions are about 14 × 14 × 10 cm, and the weight is about 250 g; (c) tilt can be measured in two directions; (d) sensors with different range and accuracy can be released; (e) the sensor can work in stand-alone mode or as a network node; (f) the sensor can be fixed to a rod to be grounded, or it can be fastened to a structure; (g) the georeferencing can be performed through the integrated GPS receiver; (h) autonomous data processing can be performed, along with (i) bidirectional data transfer.

This paper covers: (1) a description of the instrument; (2) the various hardware modules (optoelectronic level module, positioning module, data processing module, data transfer module, power supply, Frame); (3) the software implemented (tilt measurement, GPS data processing, data transfer and early warning); (4) the calibration procedures; (5) the test executed in the laboratory; (6) the first in-field data acquisitions and their discussion.

## 2. The POIS Sensor

**Figure 1:**
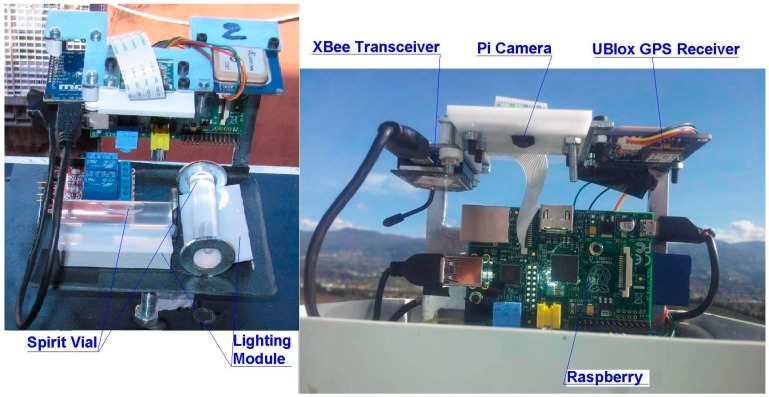
The main components of the sensor POIS.

POIS (Figure 1) is a versatile sensor, designed for the monitoring of landslide slopes and for the control of movements and tilt of structures. For this reason, several possible configurations are available, with different levels of accuracy. The sensor is basically composed by: (a) an inclination module, based on two spirit levels and a camera; and (b) a positioning module. The data processing is executed by a computer and the data transfer is performed by a transceiver, through a bidirectional protocol.

If a structure should be monitored, or a bedrock slope, for which a very rapid movement is foreseen, the positioning module is removed: in fact, the precursory movements, in this case, are smaller than the spatial resolution attainable with a cheap or medium GNSS receiver. Due to the limited predictable tilt and position variations, spirit levels with better accuracy will be used. For a structure (e.g., a retaining wall), the knowledge of the rotation center allows to detect the displacements; by using two spirit vials with a nominal accuracy (*i.e.*, the angle corresponding to a movement of the bubble of 2 mm) of 20", a minimum tilt increment of about 2" could be detected: this could allow, for a retaining wall 5 m high, to measure a horizontal displacement of about 0.05 mm at the top. In the majority of cases, the 60" spirit vials allow both good accuracy and a sufficient measurement range. To fix the sensor to the bedrock or to a structure, a metal bracket and anchor bolts are used.

For landslides which can be classified as very slow to moderate, the displacements are greater than 1 m/year [22]; in this case the spatial resolution attainable with a cheap or medium GNSS receiver is sufficient and the positioning module is foreseen. To house the sensor, a support plate is used, mounted on a pole about 2.5 m high that must be previously hammered into the ground. 

The POIS sensor can be configured as a stand-alone sensor or as a network node. Indeed, the instrument is provided with a 2.4 GHz transceiver (XBee) and with a computer, which can be programmed so as to independently perform the processing of the data collected by a single sensor or by a sensor network. Consequently, it is possible to transmit, along with the data collected by the sensors, alert signals if the thresholds determined by the monitoring center are exceeded. The bidirectional transmission also allows you to change the setting of monitoring (time of acquisition, duration of satellite acquisitions, thresholds for the observed data). 

## 3. The Hardware Modules

In the POIS sensor, we can recognize the following components: (1) the optoelectronic level module; (2) the positioning module; (3) the data processing module; (4) the data transfer module; (5) the power supply module; (6) the frame.

### 3.1. The Optoelectronic Level Module

The optoelectronic level module consists of two spirit vials, a lighting system, a camera with a five megapixel resolution and a computer for image processing. Spirit levels are generally used to set geodetic instruments plumb. These levels usually take the form of a tube-type level (toric level) for one-dimensional orientation, or a circular level for omnidirectional orientation.

For accurate measurements, circular levels are not precise enough; in this case, a couple of toric levels, mutually orthogonal, is used. Spirit levels are also used to measure small inclination angles, ranging from a few seconds to some degrees. Depending on the desired accuracy, different vials can be selected.

For measuring small inclination angles, a calibration procedure is generally performed, that makes use of a first measurement executed when the vial is in the oriented state, and of a second one made after a rotation of 180°. When it is impossible to rotate the vial, an absolute inclination respect to the horizontal direction cannot be obtained, but the tilt increment with respect to the position recorded at the moment of the installation can be measured. 

**Figure 2:**
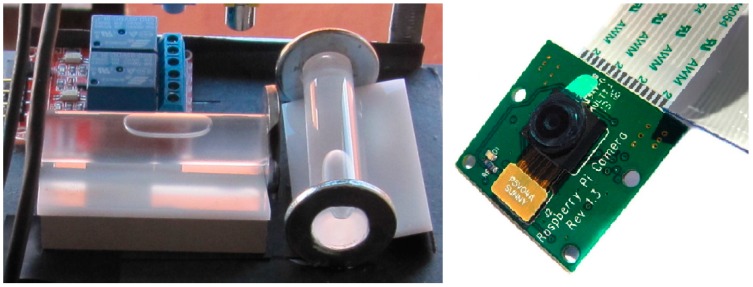
(**Left**) The spirit vials and the lighting module; (**Right**) The Pi Camera Module.

In order to determine the inclinations, images are captured of the two glass spirit level vials, arranged with mutually orthogonal axes (Figure 2 left). The images are captured using a Raspberry Pi Camera Module Rev 1.3 [23], with a resolution of five megapixel (Figure 2 right); the camera is connected to the data processing system (512 MB Raspberry Pi B). A custom software also manages the apparatus of LED lighting with diffusers. Glass bubble vials are used, having a nominal accuracy (*i.e.*, the angle corresponding to a 2 mm movement of the bubble) of 60"; since 30 pixels in the image correspond to one mm on the surface of the vial, it is possible to obtain the tilt with an actual accuracy of 0.001°, as described in the Section 5.2.

By choosing bubble vials with different characteristics, one can opt for higher accuracies and lower acquisition ranges or *vice versa*.

A high definition camera module has been chosen, compatible with the Raspberry Pi model A and model B. It provides high sensitivity, low crosstalk and low noise image capture in an ultra-small and lightweight design. It is a five megapixel camera that supports 1080p30, 720p60 and VGA90 video modes, as well as stills capture. The camera module connects to the Raspberry Pi board via the CSI connector designed specifically for interfacing to cameras. The CSI bus is capable of extremely high data rates, and it exclusively carries pixel data to the BCM2835 processor. A useful characteristic of this camera is that there are numerous third-party libraries built for it, including the Picamera Python library [24].

### 3.2. The Positioning Module

**Figure 3:**
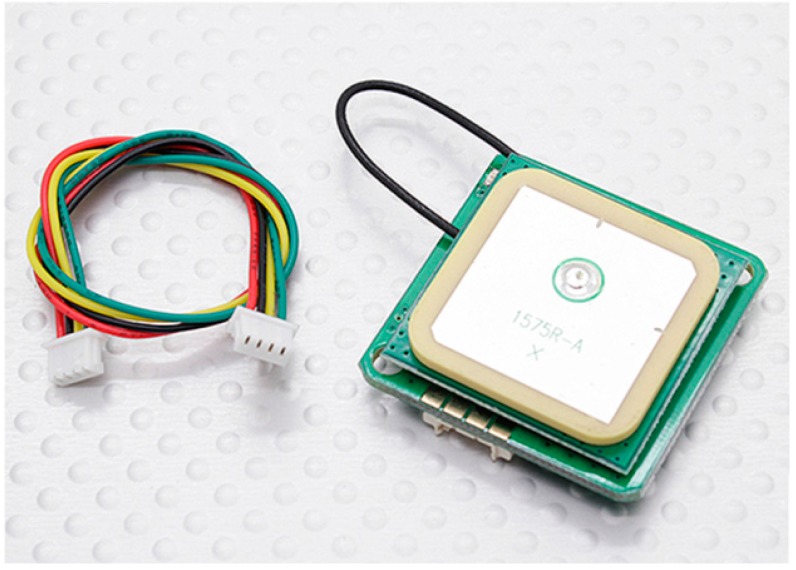
The UBlox 6M GPS module.

The positioning is performed via a GPS receiver. A Ublox NEO-6M module has been used (Figure 3), able to receive the EGNOS ionospheric corrections; the absolute positioning is obtained with a planimetric accuracy of about one meter and a height accuracy of about 2.5 m.

It should be underlined that the purpose of the acquisitions is not a satellite point positioning, but the detection of any morphological changes of the monitored surface. This is obtained by checking the changes in the baselines—mutual distances between the different sensors—computed for acquisitions repeated in almost identical terms; this way accuracy of a few decimeters can be achieved. All the sensors installed on a landslide zone are considered as vertices of a network, whose geometric variations should be computed. For this aim, a sensor is considered as a reference, and it is provided with an XBee module acting as coordinator. In this node the data acquired from the receivers converges and the calculation of the baselines is performed. 

### 3.3. The Data Processing Module

**Figure 4:**
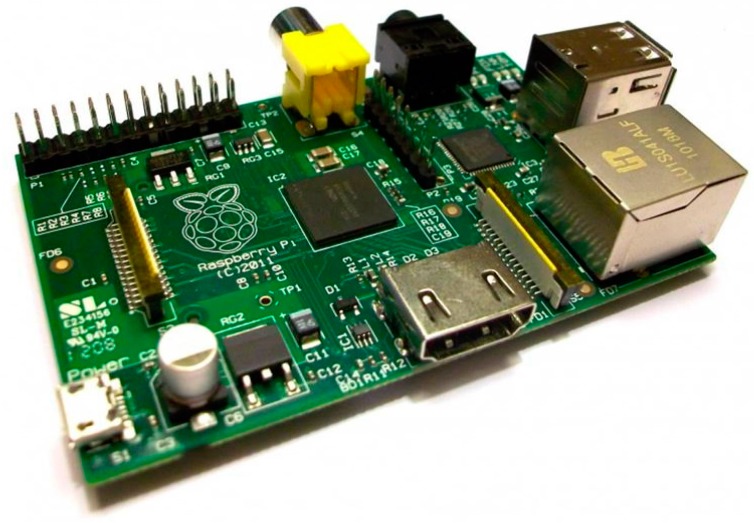
The Raspberry Pi computer.

The data processing system is constituted by a Raspberry Pi B 512 MB (Figure 4). It is a credit card-sized single-board multimedia computer, based on a processor ARM1176JZ-F at 700 MHz and a co-processor multimedia VideoCore IV, which can handle even video streams full HD. It is equipped with a 512 RAM memory and a multiformat card reader as well as a HDMI output, an Ethernet port and a small size USB. The main characteristics are given in the Table 1.

**Table 1:** Main characteristics of the Raspberry Pi computer.

Raspberry Pi	Model A	Model B
SoC (System-on-a-Chip):	Broadcom BCM2835 (CPU + GPU + DSP + SDRAM) [ 2]	
CPU:	700 MHz ARM1176JZF-S core (ARM11 family)	
GPU:	Broadcom VideoCore IV, OpenGL ES 2.0, 1080p30 H.264 high-profile decode	
Memory (SDRAM):	256 Megabytes (shared with GPU)	256 or 512 Megabytes (shared with GPU)
USB 2.0 ports:	1	2 (via the on-board USB hub)
Output video:	HDMI composite video via RCA jack	
Output audio:	3.5 mm jack, HDMI	
Onboard Storage	SD/MMC/SDIO card slot	
On-board network:	None	Ethernet 10/100 (RJ-45)
Low level peripheralsd:	2 × 13 header pins for GPIO, SPI, I^2^C, UART, +3.3 V, +5 V	
Real-time clock:	No clock or battery	
Power ratings	300 mA, (1.5 W)	700 mA, (3.5 W)
Power source	5 V via MicroUSB o GPIO header	
Size	85.60 mm × 53.98 mm (3.370 inch × 2.125 inch)	
Operating systems supported	Debian GNU/Linux, Fedora, Arch Linux [ 3] e Gentoo	
O.S. not supported:	RISC OS (shared source)	

### 3.4. The Data Transfer Module

**Figure 5:**
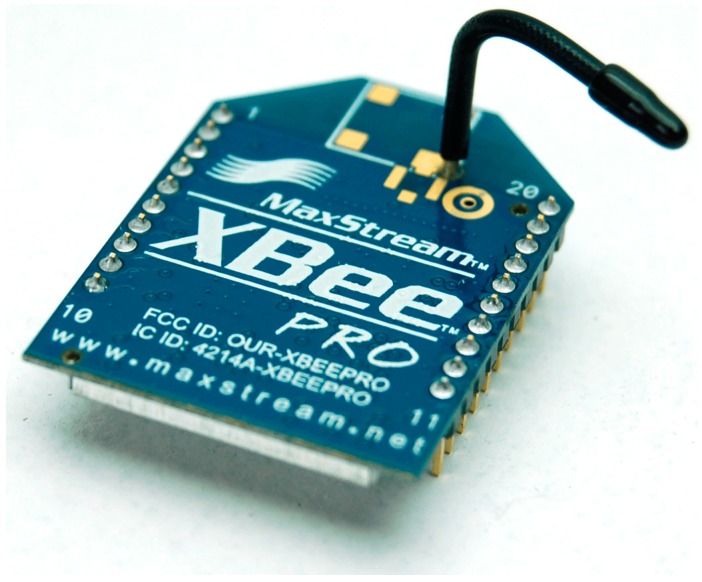
The XBee Transceiver Module.

All instruments are equipped with a 2.4 GHz XBee module for data transmission and for network configuration (Figure 5). The main characteristics are: (a) The XBee module is bidirectional: a single module is able both to receive and transmit; (b) An XBee module can be addressed unequivocally: indeed, each XBee has a serial number that distinguishes it from all other XBee. Through a simple utility it is possible to initiate communication between two modules with each other without interfering with other modules present in the surroundings; (c) It is possible to assign different channels to the modules: this minimizes even more the interferences; (d) One can use a performing antenna (wire antenna) that allows the connection up to 2 km away; (e) The cost is affordable and it is possible to create networks [25,26].

An instrument network uses the ZigBee protocol and is characterized by a set of distributed sensors, connected to a point for data collection, where a so called coordinator node is located. The XBee module is driven by the Raspberry computer, that constitutes the set of computational resources in order to perform the correlations and the processing of data, status monitoring, communication with the Monitoring Center, *etc.*

### 3.5. The Power Supply

The integrated sensor requires a power supply, which must guarantee a continuous operation in landslide zones, where it is often difficult to replace the batteries and it is impossible to connect to the electrical grid. The power supply device used in the first tests is a 17,500 mAh lead acid battery that provides all sensors with electric power. A switching between sleep and active modes was implemented. Taking into account a working time of 20 s every hour for the tilt module (camera, lighting, image processing and data transmission) and a working time of 30 min a day for the GNSS module (two sessions of 15 min every 12 h), one can foresee a life of three days for a fully charged battery of this type. The implementation of more effective power-saving strategies is planned for future development. The batteries can be recharged by electrical grid, if available, but it has been chosen, as default charging method, a photovoltaic solar panel with nominal power of 20 W.

### 3.6. The Frame

**Figure 6:**
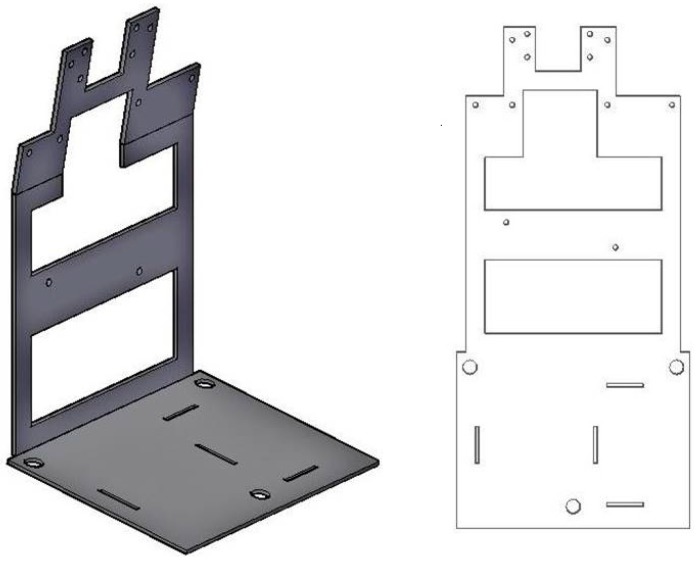
(**Left**) The frame plate partially folded; (**Right**) the drawing of the frame plate.

For assembling all components of the integrated sensor, a frame has been designed (Figure 6).

The requested characteristics are (a) the possibility of easily assembling, disassembling and accessing the components; (b) a good rigidity in order to ensure a constant distance between camera and spirit vials, necessary to have a good focusing and a constant frame scale; (c) limited weight and (d) limited dimensions.

The peculiarity of the frame is that of being made using a single piece folded and with openings that make it lighter without compromising the rigidity (Figure 6). There are slots for all the elements to be inserted (GNSS receiver, XBee, Raspberry, camera, power unit, spirit vials, *etc.*). The assembled instrument, finally, is inserted in a PVC box that ensures simple installation of the sensor in the area to monitor and weather protection (IP 65). The sensor can be coupled to a metal support that can be fixed both to a structure to be monitored, either on the support pole to be driven into the ground. Three bolts provided with levelling nuts, allow to center the bubbles of the spirit vials for the first installation.

## 4. How the Sensor Works

### 4.1. The Optoelectronic Level

**Figure 7:**
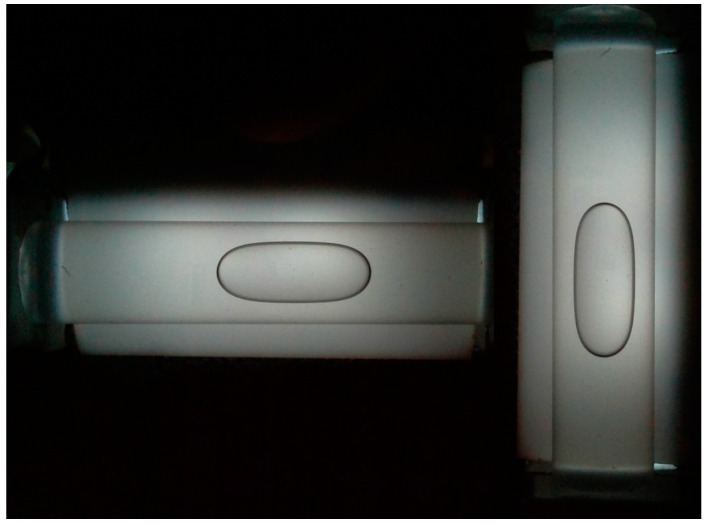
The full frame obtained by the Pi camera.

The images acquired by the camera (Figure 7) are used by a computer code, written in C++, realized on purpose. Depending on the mutual position of vials and camera, a portion of frame is considered for each vial, in order to limit the processing to a small image. 

In a first step, the edges of the bubble are coarsely obtained, considering the grey values of pixels. The candidate edge points are identified by using a threshold value. Subsequently, by interpolating these points with the least squares method, the reconstruction of the edge is performed. The interpolating curve must be a spiric section, defined as the curve of intersection of a torus and a plane parallel to its rotational symmetry axis. In our case the torus is the internal surface of the vial.

The curve is of the fourth order and its equation is [27]:

(1) $${\left(r^{2}-a^{2}+c^{2}+x^{2}+y^{2}\right)}^{2}=4r^{2}\left(x^{2}+c^{2}\right)$$

where, by assuming the reference system with the origin at the center of the torus, *y* is the rotational axis of the torus, *c* is the *z* coordinate of the plane section, *x* is the axis which completes the reference triad, *r* is the radius of the axis of the torus, a is the radius of the circle that generates the torus.

Theoretically, the only unknown is the ordinate of the plane section *c*, which varies with the temperature. If we take into account the working tolerances of the glass vial, the radii *r* and a cannot be considered fixed: thus, all coefficients of the equation should be considered as unknown values, which must be determined by the least squares method.

**Figure 8:**
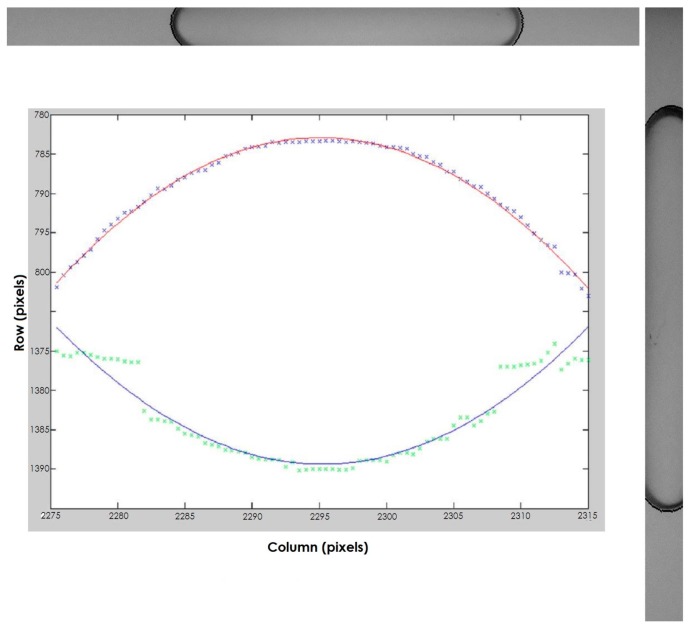
The portions of frame used for the measurement and the interpolating spiric sections obtained for the right vial.

In Figure 8, the results obtained by applying the described method to the right image are shown. We can observe that the lower edge is reconstructed, in spite of the low quality of the points obtained in the first step. The outliers were due to the inconstant level of lighting. For this reason, the power source has been stabilized and the lighting has been obtained by using a row of LED, and a polycarbonate diffuser.

### 4.2. GNSS Acquisitions

A computer code has been set up for the management of the GNSS receivers and for the data processing. The first feature of the code is to manage start, stop and sampling rate of the GNSS acquisitions. Both geographic and Earth-Centered Earth-Fixed (ECEF) orthogonal coordinates can be used. For landslide monitoring purposes, in our case, the use of the satellite receiver is foreseen for the determination of morphological variations of the surface to be monitored, rather than for single point positioning: it should be performed, therefore, the resolution of a network and the calculation of baselines.

Thus, one node is considered fixed and it houses, along with the Raspberry computer, the XBee module acting as coordinator. The coordinator, generally positioned outside the unstable zone, collects the data acquired by all receivers, while the data processing module stores the coordinates of all sensors in a matrix. The Euclidean distances between all couples of nodes are computed and compared with the ones obtained in the previous acquisitions. This way, the morphological variations of the sensors network are obtained.

To obtain the most accurate results, we follow a procedure based on the following remarks: the single point positioning error is mainly due to atmospheric errors and multipath; it also depends on the antenna quality. Given that the used receivers acquire the EGNOS signal and the mutual distances don’t exceed a few hundred meters, we can consider the atmospheric errors almost equal for all sensors. Thus the difference in the point positioning error should mainly depend on multipath and antennas. Depending on the morphology of the terrain around each sensor, the multipath effects can be quite different for each receiver, but, for the same receiver, it should be almost equal for the acquisitions performed with the same satellite configuration. This happens twice a sidereal day (23.9344696 h), so, by performing the acquisitions every 12 sidereal hours (or a multiple thereof) we can minimize the variations of the computed baselines. This way, a few decimeters error in the baseline variations could be achieved. 

### 4.3. Data Transfer and Early Warning 

The POIS sensor can be configured as a stand-alone instrument, or a network of instruments can be realized. In the first case, data transfer to the Monitoring Center is performed via the XBee module, if the distance is less than one km; for longer distances, a General Packet Radio Service (GPRS) module should be used.

When a network of sensors is realized, an XBee module acts as coordinator and as interface with the Monitoring Center. The coordinator rules the activation of the other sensors, and collects the data acquired. Its Raspberry Pi computer is programmed to perform the processing of the GNSS acquisitions. Depending on the instructions given by the Monitoring Center, the thresholds are fixed for the baselines variation and for the tilt increment. If the difference between measurements in later times exceeds one of the predetermined thresholds, an alert is sent to the Monitoring Center, along with the recorded acquisitions.

The Monitoring Center can send the commands for: (a) repeating the measurements or (b) changing the frequency of the Tilt measurement or (c) changing the sampling rate and the duration of the GNSS acquisitions. If necessary, an early warning procedure can be activated.

## 5. Calibration

### 5.1. Camera Calibration

**Figure 9:**
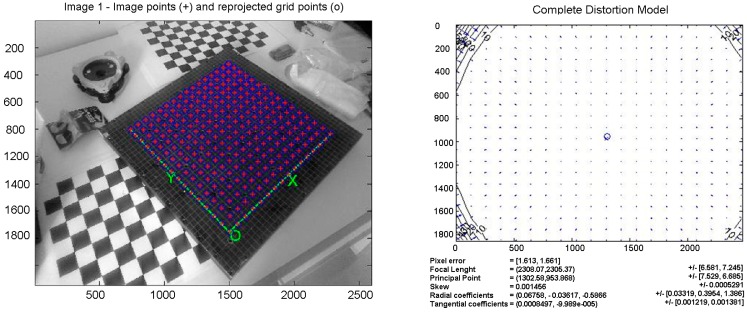
(**Left**) The crosses of the calibration grid are automatically recognized by the calibration software; (**Right**) the distortion model of the calibrated camera. The coordinates and the results are in pixels.

For the calibration of the camera, it was realized an upgrade of a well-known procedure [28,29,30], developed using Matlab^®^. The procedure can be applied to any digital camera. A calibration plate with reference points has been used. The accuracy of the points on the calibration plate is 0.1 mm. As can be seen in the left side of Figure 9, the software recognizes the points of intersection. This is done automatically, but it is foreseen the possibility of intervention by the operator that can correct any errors or eliminate false positives identified by the automatic procedure.

The main parameters of a tested camera are shown in the right side of Figure 9. The results obtained are: focal length, principal point, skew, radial and tangential distortion parameters.

### 5.2. Tilt Module Calibration

**Figure 10:**
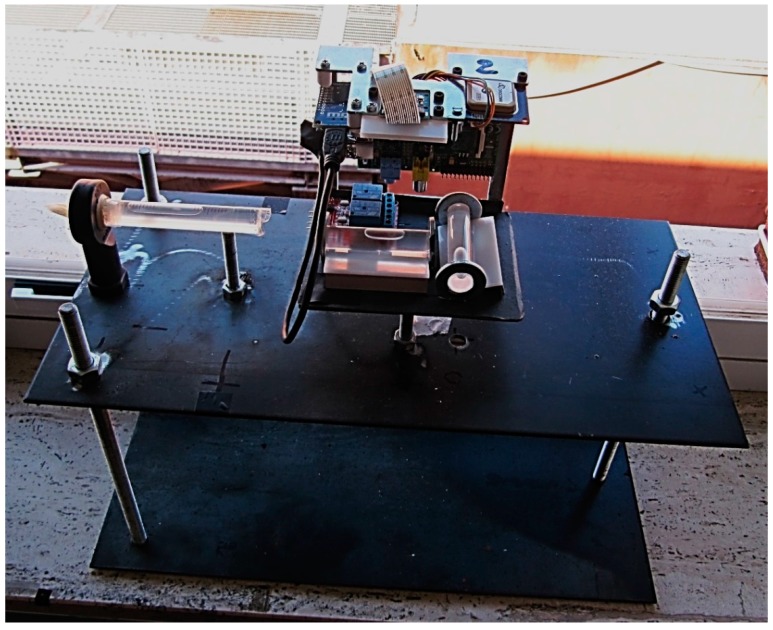
The spirit level comparator.

For the tilt module calibration, a spirit level comparator, expressly realized, has been used. The comparator (Figure 10) is constituted of a reinforced plate that houses the sensor to be calibrated and a graduated spirit vial used as reference. Both sensor and reference vial are equipped with elevation bolts. The accuracy of the reference vial, *i.e.*, the angle corresponding to a movement of the bubble of 2 mm, can be chosen depending on the accuracy of the vial to be calibrated; in the Figure 10, the vial to be calibrated has an accuracy of 60" and the accuracy of the reference vial is 2". The sensor can be positioned in two orthogonal positions, in order to perform the calibration for both longitudinal and transversal vial. A micrometric thread bolt has been installed at one end of the plate to generate tilt. The vertical displacement generated by a one-pitch revolution of the micrometric thread bolt is 1 mm, so that a rotation of one degree of the bolt causes a vertical displacement of the end of the plate, of 0.0028 mm. Thus, since the length of the plate is 55 cm and 1 rad = 206265", a rotation of one degree of the bolt causes an increment of inclination of the plate equal to (0.0028/550 × 206265) = 1.05". Given that 30 pixels in the image correspond to one mm on the surface of the vial, it is theoretically possible to detect a tilt variation of 1”. 

At the beginning of the calibration procedure, by using the elevation bolts, the bubble of the longitudinal vial is positioned at one end, whilst the bubble of the reference vial is centered and the “zero” position is read. By turning the micrometric thread, an inclination is generated, which value can be read by using the reference vial; in our case, the increment of inclination between two consecutive acquisitions was set to approximately 4". When the bubble of the reference vial reaches the border, it is centered again through its elevation bolt, to continue the acquisitions until the other end of the longitudinal vial is reached. The procedure is repeated for the transversal vial. 

**Figure 11:**
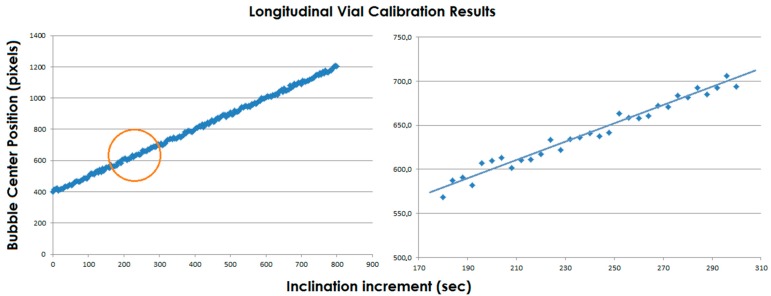
(**Left**) The results of the calibration; (**Right**) The enlargement of the circled zone and the interpolating line.

Figure 11 shows the results of the calibration for a vial. On the right side, the enlargement of the circled zone and the interpolating line are represented. The standard deviation is about 5 pixels, *i.e.*, 5".

## 6. The First Tests

### 6.1. The Lab Test

The first tests have been carried out on a group of 5 sensors positioned on the roof of the Geomatics Lab, at the University of Calabria (39°.667 N, 16°.226 E), in September 2014, for a period of 15 days. The position of the sensors has been acquired by a total station. In the lab, a permanent GNSS station is operating for seven years. 

**Figure 12:**
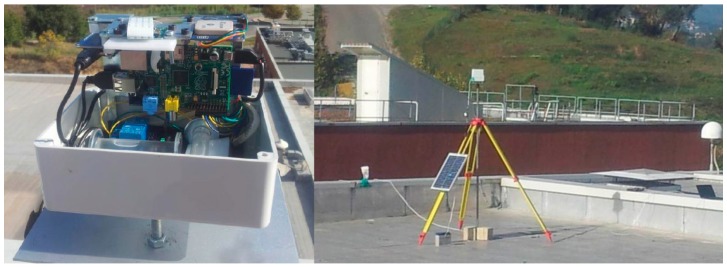
(**Left**) The sensor assembled; (**Right**) The sensor positioned on the terrace of the Geomatics Lab: the photovoltaic panel, the battery and the antenna of the GNSS permanent station are visible.

In the left part of Figure 12 we can see an assembled POIS after the centering of the bubbles of the vials and before closing the cover. On the right part one can see a sensor mounted on a pole and sustained by a tripod, along with the battery and the photovoltaic panel. The choke-ring antenna of the permanent GNSS station, housed in the lab, is visible on the right.

All sensors have acquired the tilt measurements every three hours and the position every sidereal day, with an acquisition time of 10 min and a sampling rate of 1 Hz. 

The data acquisitions carried out during the lab test confirmed the good performance of the optoelectronic level. Indeed, during the test, there were very different weather conditions, but the oscillations of the tilt measurements were limited and the standard deviation was 7".

The results of the positioning modules were compared to their actual position, derived by the total station surveying and taking into account the known position of the permanent GNSS station. All the UBlox GPS receivers showed oscillations of about 1.5 m in both North and East coordinates; the average values of the North coordinates oscillated between −0.20 m and 0.30 m, while a systematic error in the East direction, of about 1 m has been detected for all sensors. The height variations were about ±2 m. The horizontal component of the baselines, whose lengths were between 10 and 50 m, showed variations less than 30 cm.

With regard to the power supply, no problems were encountered: the lead acid batteries were recharged by the photovoltaic solar panels and the sensors were regularly powered also after four consecutive cloudy days. 

Some problems have been encountered in the data transfer: in spite of the high performing antennas we used, the connection from the roof to the lab (two floors down) was very problematic. It must be underlined that the roof floor is coated with an aluminum waterproof membrane, and that the building that houses the lab has been realized with a heavy reinforced concrete structure; in addition, in the surroundings, several data transfer devices are operating in the 2.4 GHz band. Thus, some data transfer tests have been carried out in open air, with good results up to about 1 km.

### 6.2. The Field Test

**Figure 13:**
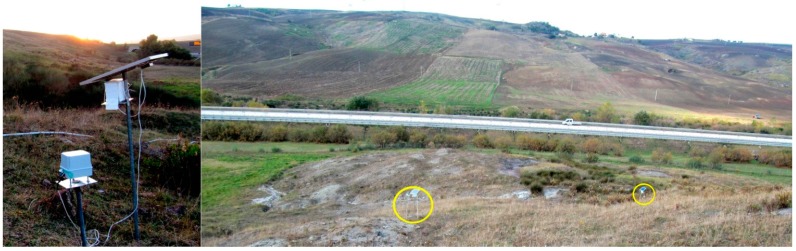
(**Left**) A POIS levelled and connected to the solar panel; (**Right**) Two sensors (yellow circles) on the toe of the monitored landslide, close to the A16 highway, near Lacedonia (Southern Italy).

The first field test began in December 2014 and it is still in progress. A network of six sensors has been installed on a landslide, at km 112 of the highway A16 (Latitude 41°.089 N, Longitude 15°.377 E). The landslide falls within the debris flow category, and the soil is predominantly coarse, thus the sensors have been fixed to supporting plates, mounted on iron poles 2.5 m high, previously hammered into the ground. Due to the absence of electrical grid, the sensors are powered by batteries, recharged by solar photovoltaic panels with a nominal power of 20 W (Figure 13).

**Figure 14:**
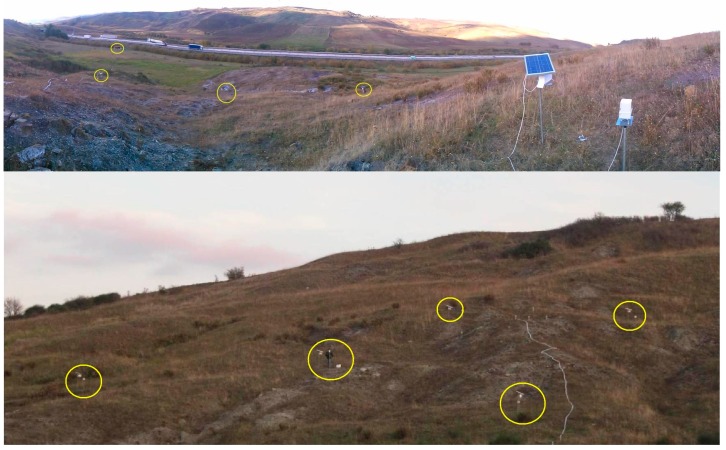
(**Top**) A partial view of the sensor network installed near the highway A16. The sensors are circled; the upper left sensor, out of the accumulation zone of the landslide, acts as the coordinator node; (**Bottom**) The sensor network seen from below. The cable on the right, powers a group of MEMS sensors installed in the same zone.

Five sensors have been installed on the accumulation zone of the landslide, while the sixth one is positioned near a cabin that houses other monitoring instruments, and it acts as coordinator (Figure 14); the length of the baselines ranges between 20 and 52 m. The landslide is monitored also by other groups, in the framework of a research funded by the E.U. [31]. In particular, a network of MEMS accelerometers has been installed near the sensors POIS; the results obtained by the two systems will be compared at the end of the experimental activities in progress.

For the data acquisitions of all sensors, the same frequency and duration of the lab test have been adopted. The results obtained after the first month of operation, suggest the considerations we report in the following.

No noticeable tilt variations have been detected by the optoelectronic levels; the oscillations of the measurements were limited and the standard deviation was about 7", like in the lab test. Some errors, however, are noted; this happens in the early morning, after two or more cloudy days, and it can be explained by insufficient lighting of the vials, due to an incomplete recharging of the batteries. Actually, in winter there are fewer hours of sunshine than in September; furthermore, the solar energy collected in the Apennine zone, where the landslide is located, is less that in the climate zone of the University of Calabria. The main problem to be solved to address this issue thus seems to be the low charging rate of the batteries.

With regard to the GPS measurements, the results obtained during the lab test have been confirmed. The horizontal distances among the five sensors sited in the landslide zone, show variations of less than 30 cm. The height variations are less than 1 m. The performances of the XBee transceivers are satisfying, since data transfer with the coordinator, about 600 m away, is quite regular.

Other problems were encountered with the telephone connection, due to the frequent interruptions in the zone. This is not, in general, a problem for data collections, when real time information is not required, but it is important for the early warning activation.

## 7. Conclusions/Outlook

In this study, a tilt and position sensor, suitable for use in the monitoring of landslide and structures, has been designed and realized, by using two spirit vials, a digital camera, a credit card-sized single-board multimedia computer and a low cost GPS receiver. The instrument, equipped with an XBee transceiver, can be used stand-alone or it can be implemented as a sensor node in a monitoring network, thanks to the wireless transmission of tilt and position measurements. After the calibration procedures, a lab test has been carried out, while the first field test is still in progress. The results obtained can be summarized as follows:

By using spirit vials with a sensitivity of 60", the tilt measurements carried out during the calibration procedures, showed a standard deviation of about 5", while the results obtained in the field test have a standard deviation of about 7": this confirms the usefulness of the sensor for the monitoring of landslides. 

The position measurements confirmed that the sensor can be used for detecting morphologic variations of the surface of landslides which can be classified very slow to moderate: in fact, the horizontal components of the baselines of the monitored network, show variations less than 30 cm, while the height variations are less than 1 m.

The power supply should be improved, given that some errors found in the tilt measurements, executed after few cloudy days, must be imputed to irregular lighting of the vials.

Future studies are aimed, by the one hand, to overcome the problems that have been faced during the first tests, and on the other hand, to optimize the instrument by further reducing its dimensions, weight and power consumption, and by incrementing the positioning accuracy. To reduce dimensions, the use of a super wide-angle lens for the camera is foreseen: this will allow minimizing the focus distance. Weight could be reduced by using lithium polymer batteries, which guarantee also a faster recharge. To minimize the power consumption, the activities of the Raspberry computer will be optimized, above all during the stand-by periods. Finally, a new antenna to be used with the Ublox GPS receiver will be tested, in order to increase the precision of positioning.
